# Operationalizing the ‘pragmatic’ measures construct using a stakeholder feedback and a multi-method approach

**DOI:** 10.1186/s12913-018-3709-2

**Published:** 2018-11-22

**Authors:** Cameo F. Stanick, Heather M. Halko, Caitlin N. Dorsey, Bryan J. Weiner, Byron J. Powell, Lawrence A. Palinkas, Cara C. Lewis

**Affiliations:** 1 Hathaway-Sycamores Child and Family Services, Pasadena, CA USA; 2 Department of Psychology, University of Montana, Missoula, MT USA; 3 Kaiser Permanente Washington Health Research Institute, Seattle, WA USA; 4 Department of Global Health, University of Washington, Seattle, WA USA; 5 Department of Health Policy and Management, University of North Carolina at Chapel Hill, Chapel Hill, NC USA; 6 Department of Children, Youth and Families - University of Southern California, Los Angeles, CA USA

**Keywords:** Pragmatic, Measure, Implementation, Stakeholder

## Abstract

**Context:**

Implementation science measures are rarely used by stakeholders to inform and enhance clinical program change. Little is known about what makes implementation measures pragmatic (i.e., practical) for use in community settings; thus, the present study’s objective was to generate a clinical stakeholder-driven operationalization of a pragmatic measures construct.

**Evidence acquisition:**

The pragmatic measures construct was defined using: 1) a systematic literature review to identify dimensions of the construct using PsycINFO and PubMed databases, and 2) interviews with an international stakeholder panel (*N* = 7) who were asked about their perspectives of pragmatic measures.

**Evidence synthesis:**

Combined results from the systematic literature review and stakeholder interviews revealed a final list of 47 short statements (e.g., feasible, low cost, brief) describing pragmatic measures, which will allow for the development of a rigorous, stakeholder-driven conceptualization of the pragmatic measures construct.

**Conclusions:**

Results revealed significant overlap between terms related to the pragmatic construct in the existing literature and stakeholder interviews. However, a number of terms were unique to each methodology. This underscores the importance of understanding stakeholder perspectives of criteria measuring the pragmatic construct. These results will be used to inform future phases of the project where stakeholders will determine the relative importance and clarity of each dimension of the pragmatic construct, as well as their priorities for the pragmatic dimensions. Taken together, these results will be incorporated into a pragmatic rating system for existing implementation science measures to support implementation science and practice.

## Background

Despite their potential for informing the practice of implementation, measures largely remain a scientific phenomenon and are rarely employed by stakeholders (e.g., providers, policymakers, etc.) seeking to make clinical program changes. This may be the case for several reasons, with two of the most critical being: (1) stakeholders typically are not trained to use quantitative measures (which may require special skills and/or knowledge to identify, select, administer, score, interpret and/or apply new knowledge from measures); (2) measures are typically not designed for use outside of the research context (e.g., high participant burden, low relevance to clinical activities, confusing/complicated scoring, high cost, etc.). Often researchers may be trying to capture or control for a number of variables within one measure, or across several measures, leading to potentially lengthy, impractical, complex measures that are not feasible for use in everyday practice. Though measures used in research may be psychometrically strong, their practical or pragmatic utility may be low. Unfortunately, without more pragmatic measures [1], stakeholders will remain limited in their ability to efficiently make decisions about implementation. For instance, measuring certain determinants of practice may inform a clinic director’s decision to invest in a particular evidence-based practice (EBP), or may help to identify that communication between care provider levels is suboptimal but key to sustaining an EBP. If instruments that measure these constructs are only accessible within the research context, the gap between implementation science and practice will continue to grow.

Intervention researchers have highlighted the clinical utility of employing evidence-based, pragmatic measures (i.e., evidence-based assessments, EBA) of health status and mental health functioning in practice settings to guide treatment decisions and improve patient outcomes. For example, the Patient Health Questionnaire (PHQ) was developed with careful consideration of pragmatic qualities (e.g., length: 2- and 9-item versions are available; cost: free of charge) to provide clinicians with a psychometrically strong, accessible measure of depression associated with recommended treatment actions based on national norms [2]. EBAs, such as the PHQ, are critical to aid in selecting evidence-based practices (EBPs) and to ensure that the EBPs are properly used and evaluated [3]. However, there has not been an explicit focus on making implementation science measures pragmatic. For instance, the Organizational Social Context (OSC) measurement system provides an assessment of organizations’ culture, climate, and work attitudes in comparison to national norms [4]. The OSC has been shown to predict implementation, service system, and clinical outcomes [4–9], and has also informed the development of an organizational implementation strategy (the Availability, Responsiveness, and Continuity [ARC] intervention) that has been shown to improve organizational context and outcomes for youth. There is mounting evidence that organizational culture and climate are critical to assess and address within both implementation research and practice. Unfortunately, despite possessing some pragmatic qualities (e.g., actionable, sensitive to change [1]), the OSC is proprietary and lengthy, which could limit its use in both research and practice.

“Pragmatic measures” is a relatively new construct, or way of conceptualizing measurement [1]. Glasgow and Riley [1] were the first to explicitly apply this conceptualization to measures in the domain of health status and mental health functioning, indicating that pragmatic measures are those that are (a) important to stakeholders, (b) low burden for respondents and staff, (c) actionable, and (d) sensitive to change. However, to our knowledge, this conceptualization was generated by the authors (who based their ideas on a convenience sample of relevant literature) in the absence of any stakeholder involvement. Although there is clear face validity to these dimensions of the pragmatic construct, particularly as it applies to measurement, it is not clear whether stakeholders would agree that these dimensions have face validity or that they are what make a measure pragmatic. It is also unclear whether stakeholders regard certain features as more important than others, or whether they identify other features as important that the authors overlooked. Without stakeholder involvement in the operationalization of the pragmatic measures construct, the impact of this work will be undermined.

We propose that evidence-based, pragmatic measures of implementation determinants (i.e., barriers and facilitators), processes, and outcomes would be useful in research and practice. That is, we are suggesting that implementation science measures could aid stakeholders in any context in prospectively assessing actionable barriers and facilitators, monitoring implementation impact and feeding back implementation outcomes. Without the development and availability of pragmatic measures for these domains, the very individuals and contexts researchers and measure developers are attempting to work with/within will be inaccessible. To ensure that measures are “pragmatic,” we aim to learn how stakeholders conceptualize pragmatic measure qualities and to construct stakeholder-informed, reliable, and valid criteria for rating the pragmatic quality of measures. The objective of the present, multi-method study was to generate a stakeholder-driven operationalization of the pragmatic construct through the completion of two aims: (1) complete a literature review to identify pragmatic measures dimensions as found in the research, and (2) conduct interviews with stakeholders to reveal unique pragmatic measures dimensions. This represents a significant step forward for the field. The resulting pragmatic rating criteria may motivate researchers to include these ratings in systematic reviews and for measure developers (whether in research or practice settings) to consider these properties in new measure creation. Comparable rating criteria have over 1000 citations, despite their relatively recent development, suggesting the potential for significant impact [10, 11]. By combining results from the literature review and interviews with representative stakeholders we will develop a more rigorous and relevant understanding of the pragmatic measures construct on which to base future work. In addition, our ultimate goal is to combine relevant pragmatic domains and dimensions with similar set of psychometric domains and dimensions to culminate in a systematic criteria/rating framework that can be used to identify and/or develop measures that are strong in both pragmatic *and* psychometric qualities.

## Methods

In order to identify the synonyms and defining features of what it means for measures to be ‘pragmatic,’ both inductive and deductive methods were utilized. First, a literature review was conducted to inductively identify synonyms, descriptors, or dimensions, of the ‘pragmatic’ construct. Following the literature review, a stakeholder interview was developed to deductively identify and refine the features of what it means for a measure to be ‘pragmatic.’ These pieces of information were synthesized and cross-walked to develop a comprehensive list of terms defining the pragmatic construct.

### Method 1: Literature review

As a first step toward identifying a list of terms related to the pragmatic construct, a review of the literature was completed. On 11/16/2015, the first author (CC) searched PsycINFO and PubMed using the search strings detailed in Table 1 to identify relevant literature. The specifier ‘NOT language’ was utilized. In the first attempt to search utilizing the term ‘pragmatic,’ it became clear that ‘pragmatic language disorder’ substantiated a majority of results, particularly within PubMed. Thus, the specifier ‘NOT language’ was added to the search string to filter articles. The second author (HH) completed a duplicate literature search on 01/20/2016. We reviewed titles and abstracts to exclude irrelevant articles and identify articles for full-text review. Full texts of articles that made it past the title and abstract review were accessed and reviewed to identify possible dimensions of the pragmatic construct. The reference lists of included articles were also used to identify additional articles. All synonyms of ‘pragmatic’ (e.g., ‘simple’) and/or potential dimension terms/phrases (e.g., ‘ease to score’) were coded, including the field from which the terms were extracted (e.g., mental health treatment outcomes, geriatric rehabilitation, pain management, etc.). Each included article was independently reviewed by the first and second authors (CS & HH) to ensure reliability of coding, and any discrepancies were discussed until consensus was reached.

**Table 1:** *Search Strings*

Level	Search terms
1	pragmatic AND
2	assessment* OR measure* AND
3	implementation NOT
4	language

All terms and phrases that were synonymous with, used to define, or apparent dimensions of the ‘pragmatic’ construct for measurement were noted. Terms/phrases were edited by the investigative team for redundancies. Lengthy phrases (i.e., longer than 4 words) were edited for parsimony. Importantly, our review could be subjective to publication bias because it only included literature published in peer reviewed scientific journals; thus, potential instruments living outside this space could have been overlooked. However, the purpose of this review method was to identify peer-reviewed articles that included specific assessment to the pragmatic measures construct.

### Method 2: Stakeholder interviews

Stakeholder interviews were conducted in order to ensure that the operationalization of the pragmatic construct was a participatory process. We also explored the stakeholders’ context, experiences, and use of measurement in implementation [12, 13]. To ensure that stakeholders from multiple contexts were able to provide perspective on ‘pragmatic’ measurement, we purposively sampled [14] and recruited seven stakeholders from multiple organization types and service roles: outpatient community mental health center, school-based mental health, state mental health department, residential center, inpatient hospital, implementation consultant for non-profits, international implementation consultant. The investigative team identified five US-based panelists representing distinctly different agency types listed above, as well as two additional international stakeholders, all of whom had been directly involved in evidence-based practice implementation. This study was approved by the Internal Review Board at Indiana University under expedited review. Informed consent was approved to be obtained verbally, and informed consent procedures were read aloud to interview participants prior to starting to record the interview (which was completed over telephone). Participants were informed that their participation was voluntary and that they may choose to not participate at any time.

The interview questions directly targeting the pragmatic construct domain were drafted in consultation with a qualitative expert (fifth author, LP). They were further edited by the investigative team, and were submitted and approved by the Institutional Review Board. The interview items targeting pragmatic measurement included exploration of what ‘pragmatic measurement’ meant to the stakeholder participants similar to synonyms found in the literature search (e.g., “What comes to mind when you hear the words, ‘practical or pragmatic measure’?”) and what attributes of measures they see as most and least relevant to the pragmatic construct (e.g., “If you have ever used any tools or measures for implementing new programs or clinical changes, what features or characteristics about those tools or measures made them pragmatic or practical?” “Have you ever used tools or measure that you did not consider practical or pragmatic? If so, what about those tools or measures made it impractical or not pragmatic?”). Interviews were conducted over a two-week period in 2016, and each took approximately one hour to complete. They were audio-recorded and transcribed using a human voice transcription service. Transcripts were checked for accuracy (CS & HH), and imported into NVivo 9 for data management and analysis.

### Process for integrating findings

Interview transcriptions were coded by the first and second authors using a systematic content analysis to identify core patterns or themes within the data. [15] Coding of transcripts was completed using a constant comparative approach, moving between the interview materials and results of the literature search to uncover similarities and differences between the various sources of information [16, 17]. Specifically, themes were first inductively coded regarding ‘pragmatic’ synonyms and/or dimensions, as well as antonyms/non-pragmatic dimensions. Next, we adopted an iterative process to integrate the systematic literature review and interview findings for the pragmatic construct. Redundant terms between the systematic literature review and interview findings were eliminated, and the investigative team (CS, HH, CD, BP, BJW, & CCL) reviewed cases where terms/phrases were similar but not exact to obtain consensus on whether to delete or reword terms. Next, the terms from the stakeholder interviews were reviewed for redundancy and if needed, stakeholder panelists were contacted again to seek clarity on their statements (two participants were re-contacted for clarifying in this way). Lastly, the inductively-developed themes from the literature review were combined with deductively-derived themes from the stakeholder interviews to create a final list of terms/phrases [18, 19]. For the final list of terms, the investigative team further refined the wording of each theme to achieve clarity, precision, and distinctiveness. We refined the terminology because it was predicted that pragmatic dimensions would include different categories or levels similar to how reliability has variations in dimensions (e.g., test-retest, inter-rater, etc.). For instance, “low burden” may include the sub-dimensions: length, scoring, and cost.

## Results

### Literature review

The PsycINFO and PubMed database searches revealed 198 articles and book chapters containing the search string criteria in the title or abstract. Titles and abstracts were reviewed for all results. Of the 198 articles, only 10 (5%) contained a definition or described characteristics of ‘pragmatic’ as an assessment construct and were thus included in our review. Importantly, three of the 10 articles described characteristics of ‘pragmatic’ clinical trials, but the focus of these works were on effectiveness vs. efficacy trials and research design frameworks for implementation, not measurement; thus, these articles were excluded. An additional article describing characteristics of ‘pragmatic’ as an assessment construct was identified during the duplicate literature review, increasing the total sample size to eight articles.

Multiple terms/phrases that were related to or synonymous with ‘pragmatic’ or practical measurement were extracted from the final eight articles. The articles contained terms/phrases that crossed five different areas: treatment outcome research (*N* = 3 articles); geriatric rehabilitation (*N =* 1 article); pediatric psychopharmacology (*N =* 1 article); pain management (*N* = 1 article); implementation science (*N* = 1 article); biomedical research (*N* = 1 article). The full list of terms/phrases can be found in Table 2.

**Table 2:** *Pragmatic Literature Review Terms*

Author (Year)	Scientific Discipline	Terms Relevant to the Pragmatic Construct
Pfieffer (1996)	Treatment Outcomes	confirm efficacy of intervention
		efficient
		lead to treatment planning
		low complexity
		low cost
		provide a clinical cut-off score
Slade (1999)	Treatment Outcomes	able to be photocopied
		acceptable
		allows feedback data to be returned in a positive way
		available
		benefits outweigh costs
		brief
		compliments clinical judgment
		contains readable indices
		easily accessible
		easy to use
		feasible
		flexible administration
		free
		low administration time
		meaningful for use in typical clinical settings
		non-duplicative
		offers relative advantage
		provides meaningful feedback
		provides understandable data to provider and patient
		reduces response bias related to social desirability
		relevant
		simple
		suitable for routine
		sustainable
		valuable
Anderson (2003)	Treatment Outcomes	brief
		demonstrates functional improvement
		easy to administer
		easy to score
		focused
		provides acute, clinically relevant information
Glasgow (2013)	Treatment Outcomes	actionable
		broadly applicable
		feasible for use in real world settings
		important to stakeholders
		psychometrically strong
		related to interventions
		related to theory or model
		relevant
		sensitive to change
		unlikely to cause harm
		used for benchmark
Auger (2006)	Geriatric Rehabilitation	able to detect clinically important change
		acceptable
		age appropriate
		applicable
		available
		clinically relevant
		compatible format
		easy to administer
		easy to use
		feasible
		identifies client difficulties
		low administration time
		low completion time
		low cost (administration, scoring, materials)
		low examiner burden
		low respondent burden
		offers clinical usefulness/utility/value
		practical
		provides clear instructions
		provides meaningful score distribution
		sensible
		short completion time
		significant assessment content
		simple presentation/administration
		useful for intervention planning or process
		useful for setting
		valid
Coghill (2011)	Pediatric Psychopharmacology	health-related quality of life measures
		impairment measures
Gelinas (2008)	Pain Management	accessible (allowed to be used)
		can be easily applied in clinical settings
		easy to complete
		feasible
		high clinical utility
		meaningful
		provides results that are useful in clinical setting
		quick to use
		results optimize care and patient outcomes
		simple to understand
		supports clinical practice and decision making to optimize care and patient outcomes
Kroenke (2015)	Biomedical Research	accessible/free/in the public domain
		available in different languages
		brevity of items
		can be administered electronically
		can be administered to vulnerable populations
		can be downloaded from the internet
		cross-cutting (used for multiple diseases/conditions)
		efficiently informs specific actions that will be embraced by practitioners
		includes easy-to-remember cut points
		leads to greater detection of problems in sensitive areas
		length, specificity, and granularity are suitable for the setting
		minimizes clinician or interviewer bias
		minimizes respondent burden
		minimizes time the clinician needs to devote to data collection
		multipurpose (screening, diagnosis, monitoring)
		provides score easily interpreted by clinicians and patients
		reliable and valid self-administration
		scores facilitate communication and decision-making
		scores guide diagnostic or therapeutic action/decision making
		simple/easy to score

There were several redundancies in terms across the eight articles, as well as terms/phrases with very similar wording. Therefore, terms/phrases were edited for redundancies and length (phrases were edited to be no more than 4 words in length), resulting in a final list of 37 terms/phrases related to or synonymous with ‘pragmatic’ or practical measurement. Example terms/phrases include measures that are ‘simple,’ ‘easy to administer,’ and ‘brief.’

### Interviews

The average age of the seven stakeholder panelists was 51.8 years (57% Female, 100% Caucasian). Three stakeholders held doctoral degrees, three held a master’s degree, and one held a bachelor’s degree. Stakeholders’ specific settings included a community mental health center (primarily residential), state Departments of Education and Mental Health, an outpatient community mental health center, and two different hospital-based programs (one with an area of emphasis in substance abuse).

Qualitative coding results from the stakeholder panelist interview designed to gather information about how to define ‘pragmatic’ characteristics of assessment measures produced 39 domains of pragmatic measures (e.g., cost), 11 dimensions of those domains (e.g., less than $1.00 per measure), and 16 antonyms of what a pragmatic measure is (e.g., costly) (see Table 3). Fourteen terms/phrases were identified by the research team as being ‘actionable’ terms, such that the term/phrase implied linkage with a specific action, intervention, or decision (e.g., “provide a cut-off score leading to an intervention or treatment plan”). Seven items were cut from the domain list by the investigative team because they only related to the ability of a measure to inform clients about their clinical outcomes. Though clinical outcome measures may be the most likely adopted measures within organizations implementing EBPs, the concern was that including these measures would cause participants to focus on clinical outcomes exclusively rather than implementation in general in future research tasks (e.g., “further them [clients] in the discussion about why they are seeking treatment”). A final list of 66 terms were derived from the stakeholder interviews.

**Table 3:** *Pragmatic Interview Terms*

Domain (Synonym)	Dimension	Antonym
accessible	easily understood by client	requires an explanation to be understood or responded to
accessible language	language is clear	wordy
allows an organization to readily assess progress over time ^a^		
believed in it		
can be done over the phone		
can be extracted from EMRs		
choosing		
clinical/client progress ^a^		
connect to outcomes ^a^		
conveys information to help people understand where they are in the system ^a^		
cost	less than $1.00/measure	costly
don’t have to be an expert	can be given to someone else to follow through	requires an additional set of skills or expertise to utilize it
		requires a huge amount of training
easy to use		burdensome
		cumbersome
feasible		interrupts daily work flow or productivity
fits within the sphere and scope of activities that are done by an organization		structures their work
further them in the discussion about their symptom ^a^		
further them in the discussion about what it is they want to work on ^a^		
further them in the discussion about why they are seeking treatment ^a^		
gives suggestive decisions about next clinical step ^a^		
important to clinical care	explain and utilize measure in session	not relevant to treatment
	part of routine clinical work	
	not for quality improvement only	
informs decision making ^a^		
intuitive		
meaningful		tedious
measurable	specific for a given time frame	
natural output of clinical work and quality improvement activities		mimics their work
provides suggestions for clinicians about adherence or fidelity ^a^		
responsive		
results help reduce people from going to higher level of care ^a^		
sensitive to change		
short	less than 15 min	long
		time consuming
successfully measures things for you that are not working ^a^		
tells you what clinical interventions work for certain types of behaviors ^a^		
thought out		unrefined
tied to reimbursement		
transparent	transparent up and down a system	subjective
useful results	reliable and valid results	
uses an easy scale		
valuable to the clinical process ^a^		
works for context		

^a^Denotes terms that are related to an ‘actionable’ feature

### Integrated results

The terms/phrases from the literature review and stakeholder interviews were combined to construct a final list. Items that were thought to be confusing or duplicative were deleted (e.g., “choosing” was deleted due to lack of clarity on this term), and phrases that were more lengthy were edited to be approximately four words or less (e.g., “Fits within the sphere and scope of activities that are done” was edited to “Fits organizational activities”). The final list consisted of 47 terms/phrases related to pragmatic measurement (see Table 4). Table 4 outlines the consensus items – those terms/phrases that were found in both the literature review and the stakeholder interviews, as well as terms/phrases that were unique to either literature review or interviews. Notably, there were nearly as many items identified by researchers and found only in the literature review as there were consensus items. In addition, stakeholders reported an additional ten terms/phrases not found in the literature review.

**Table 4:** *List of Final Pragmatic Terms/Phrases*

Dimension	Identified Through:		
	Lit Review and Interviews	Lit Review Only	Interviews Only
Brief	✓		
connects to clinical outcomes	✓		
creates low assessor burden (ease of training, scoring, administration time)	✓		
easy to use	✓		
feasible	✓		
fits organizational activities	✓		
important to clinical care	✓		
informs clinical intervention selection	✓		
informs decision making	✓		
low burden	✓		
low cost	✓		
meaningful	✓		
not wordy	✓		
offers a compatible format to setting/user	✓		
produces reliable and valid results	✓		
reveals problems/issues in process or outcomes	✓		
sensitive to change	✓		
simple	✓		
the output of routine activities	✓		
acceptable (to staff and clients)		✓	
applicable		✓	
confirms efficacy of interventions		✓	
creates a low social desirability bias		✓	
easy to administer		✓	
easy to interpret		✓	
easy to score		✓	
efficient		✓	
focused		✓	
generates data that provides a positive feedback loop (not used for staff punishment)		✓	
has a meaningful score distribution		✓	
non-duplicative		✓	
of low complexity		✓	
offers flexible administration time		✓	
offers relative advantage over existing methods		✓	
optimizes patient care		✓	
provides a cut-off score leading to an intervention or treatment plan		✓	
relevant		✓	
accessible by phone			✓
assesses organizational progress over time			✓
completed with ease			✓
informs adherence of fidelity			✓
intuitive			✓
offer automated scoring or can be scored elsewhere			✓
requires no expertise			✓
tied to reimbursement			✓
transparent			✓
uses accessible language			✓

## Conclusions

The current study focused on developing a comprehensive stakeholder-driven operationalization of the pragmatic construct for implementation measurement. Two different methods were used. The literature review phase of the current project revealed that research studies referencing the pragmatic qualities/aspects of measurement were relatively rare, with only eight studies from which to extract data. Importantly, work by Glasgow and colleagues [1, 20] and Kroenke and colleagues [2] were explicit in their attempts to describe pragmatic measure criteria and serve as the basis for the initial steps of our work. However, their methodology was similar in that it involved primarily literature reviews, authors’ perspectives (i.e., expert review), and face validity of these features. To our knowledge, no studies have merged these methodologies with stakeholder interviews. Without the stakeholder perspective, researchers and measure developers risk building non-implementable, unsustainable measurement models for their work, and stakeholders risk being unable to demonstrate effective outcomes or understanding predictors of their own implementation efforts. Directly incorporating the perspectives of stakeholders who would be utilizing pragmatic measures seems to be a critical next step in the research.

Results from the current study revealed that while there is significant overlap of terms related to the pragmatic construct in the existing literature and from stakeholders’ interviews, a number of terms were unique to each methodology. For instance, only the literature review revealed that the ability of a measure to be used for benchmarking would be relevant to its pragmatic features; whereas, only stakeholders suggested that a pragmatic measure would be one that could be integrated with an electronic medical/health record. This finding underscores the importance of surveying stakeholders for their perspectives given that many domains and dimensions would have been missed otherwise. Indeed, understanding the characteristics of pragmatic measures from stakeholders’ perspectives serves as a first step in the process of also learning about which characteristics they value or believe are most important relative to measures being pragmatic. Research on providers’ attitudes toward clinical assessment measures has shown that attitudes can vary significantly based on the practical characteristics of measures [21]. Thus, increasing the relevance, practicality, and applicability of measures will be imperative for their use in implementation initiatives.

Importantly, 14 terms/phrases were indicative of measure characteristics that describe an ‘actionable’ criterion (e.g., ‘informs decision-making’). That is, stakeholders suggested that pragmatic measures should enable some direct course of action such as the selection of a particular intervention. Indeed, stakeholders’ use of measures likely hinges on whether measure results provide clear information on specific decisions, interventions, or policies. Though scarce, research in the area of pragmatic measurement has included ‘actionability’ as an important criterion in defining the pragmatic construct [1]. Therefore, it may not be sufficient for stakeholders to learn about the level/magnitude/presence of a particular determinant of practice (e.g., implementation readiness) but that measure results would provide guidance regarding whether or not to pursue one implementation strategy over another given the level/magnitude/presence of the identified determinant, for example. It is our concern that implementation science measurement is quite far from achieving this purpose. In addition, understanding the dimensions of the criteria that were identified in the initial phases of this work will be an important next step. For instance, ‘low cost’ was identified as a domain in both the literature review and stakeholder interviews but it is still unknown how stakeholders would further define as ‘low’ in cost (e.g., less than $1.00 per measure?). Our team will empirically evaluate all known measures of constructs included in the Consolidated Framework for Implementation Research [22] and the Implementation Outcomes Framework [23] to ascertain their pragmatic *and* psychometric strength in a later phase of this National Institute of Mental Health funded work [24].

### Limitations

The current study was not without limitations. As stated previously, the literature review was subject to bias given that only published literature was assessed; thus, terms related to the pragmatic construct that may fall outside of this source could substantially change the results of the systematic review. However, it was with intention that published articles were first assessed, coupled with stakeholder interviews to thoroughly capture both empirical and colloquial terminology. Also, settling on the use of term ‘pragmatic’ to define the construct as it applies to measurement was based on the small, but exiting, literature by Glasgow and colleagues regarding pragmatic measures. It is possible that one of the synonyms identified in the current study may be more broadly suited to define the construct. Given that the extant literature settled on this terminology, however, we continued with it as it seemed the most appropriately suited label for the construct.

In addition, though saturation appears to have been achieved, only seven stakeholder participants were recruited and it is possible that if more stakeholders, or individuals in different professional roles, were recruited that additional pragmatic criteria may have been identified. In addition, our international stakeholder group did not include individuals from low- and middle-income countries (LMIC), and therefore our representation may be lacking or may impact how the pragmatic construct is defined with respect to measurement in these contexts. Further investigations in this area should attempt to include LMIC representation and/or combine this work with other investigators researching similar concepts in these areas. Further, as the current study was an investigation of the nomenclature associated with pragmatic measurement, which resulted in multiple terms, it remains unclear which pragmatic criteria stakeholders would regard according to relative importance. This limitation will be addressed in future phases of this work with concept mapping and Delphi methodologies.

In summary, the current study involved the development of a stakeholder-driven operationalization of the pragmatic construct for implementation measures. Though there was clearly consensus between the literature review results and the stakeholder interview themes regarding what features define pragmatic measurement (e.g., ease of use, brief, results link to specific actions), there were also a number of terms identified that were unique. Thus, involving both expert opinion as well as stakeholder perspective is critical to achieving a comprehensive understanding of the defining features of pragmatic measurement. The current study was a first step in elucidating the terms/phrases associated with the pragmatic construct. The next phase of our work in defining pragmatic measurement will be to utilize the combined results of the current study in a concept mapping process to determine the categorization of terms followed by a Delphi activity to obtain their relative importance to one another. By doing this, the investigative team can determine which domains of pragmatic measurement appear to be most relevant *and* stable across both the existing literature and stakeholder perspectives. Future work may also consider additional empirical applications of this work, such as how certain domains may be relevant at different phases of implementation (e.g., pre-implementation vs. sustainability). The ultimate goal of our work is to put forth a reliable and valid criteria for assessing the pragmatic strength of implementation measures. By combining a full spectrum of stakeholder-driven, operationalized pragmatic criteria for implementation measurement, the field will be better informed to develop measures that are both psychometrically strong as well as pragmatic. Insuring that implementation measures are high in standardized criteria for research purposes, as well as practical, actionable, and easily palatable among stakeholders will aid in bridging the implementation science and practice gap.

## Abbreviations

ARC: Availability, Responsiveness, and Continuity
EBA: Evidence Based Assessments
EBP: Evidence Based Practice
OSC: Organizational Social Context
PHQ: Patient Health Questionnaire
